# Age‐Related Osseous Measures of the Temporomandibular Joint and Mandibular Ramus During Childhood Assessed by Magnetic Resonance Imaging

**DOI:** 10.1111/ocr.70071

**Published:** 2025-12-17

**Authors:** Juliet Zuying Shen, Maurice Molnar, Dominik Alois Ettlin, Daniel Beat Wiedemeier, Christian Johannes Kellenberger

**Affiliations:** ^1^ Department of Diagnostic Imaging University Children's Hospital Zürich Zürich Switzerland; ^2^ Children's Research Center University Children's Hospital Zürich Zürich Switzerland; ^3^ Department of Reconstructive Dentistry and Gerodontology, School of Dental Medicine University of Bern Bern Switzerland; ^4^ Center for Dental Medicine University of Zürich Zürich Switzerland

**Keywords:** age‐related normative values, children, magnetic resonance imaging, mandible, temporomandibular joint

## Abstract

**Objective:**

This study aimed to provide age‐related osseous measures of the temporomandibular joints (TMJs) and mandibular ramus in asymptomatic children and adolescents and to develop percentile reference data for ramus height.

**Material and Methods:**

A retrospective cross‐sectional study was conducted on 133 asymptomatic participants (67 males, 66 females; age range 0–19 years) who underwent magnetic resonance imaging of the head, including the TMJs and posterior parts of the mandible. Mandibular ramus height, glenoid fossa depth, articular eminence inclination angle, and condylar dimensions (mediolateral and anteroposterior) were measured. A single observer assessed 266 TMJs, and a second observer repeated measurements for 80 TMJs to evaluate intra‐ and inter‐observer reliability using the intraclass correlation coefficient (ICC) and Bland–Altman plots. Age‐related growth curves were estimated using the Box‐Cox Cole and Green (BCCG) distribution, modeling median, coefficient of variation, and skewness as cubic spline functions of age. The optimal spline degrees of freedom were selected using the Bayesian information criterion. Percentile curves were derived, and biannual lookup tables were provided.

**Results:**

Ramus height, glenoid fossa depth, articular eminence angle, and condylar width increased with age, whereas condylar depth exhibited variable patterns during adolescence. Intra‐observer reliability was excellent for all measurements. Ramus height and condylar width measurements demonstrated excellent inter‐observer reliability, while glenoid fossa depth, articular eminence inclination angle, and condylar depth measurements showed good inter‐observer reliability.

**Conclusion:**

This study provides age‐related osseous measures of the TMJs and mandibular ramus in asymptomatic children and adolescents aged 0–19 years. The percentile curves for mandibular ramus height may aid in assessing growth in children with juvenile idiopathic arthritis for monitoring disease activity and treatment responses.

## 1 Introduction

The temporomandibular joint (TMJ) is a synovial joint formed by the connection between the skull base and mandibular condyle. It comprises a biconcave articular disc, joint capsule, synovial fluid, articular surfaces, synovial membrane, and cartilage [1]. Various ligaments and muscles are attached to this anatomically and biomechanically complex structure, providing stability and controlling mandibular movement [2].

Mandibular growth is primarily driven by endochondral bone formation at the condyles and intramembranous bone formation at the periosteum [3]. The mandibular condyle is coated with fibrocartilage [4] and serves as the most essential growth site of the mandible [5, 6]. While condylar growth contributes to mandibular elongation and rotation, intramembranous bone formation at the periosteum facilitates extensive surface remodelling [3]. In the mandibular ramus, periosteal activity leads to bone resorption at the anterior border and bone apposition at the posterior border [7]. Changes in the length of the mandibular ramus and condylar process can mimic either normal mandibular growth or pathological TMJ processes [6].

Juvenile idiopathic arthritis (JIA) is a chronic inflammatory disease and the most common form of arthritis in children, affecting synovial joints including the TMJ [8, 9]. TMJ involvement is highly prevalent in children with JIA, ranging from 17% to 87% [9]. Since the mandibular growth sites are located at the condylar poles, synovial inflammation leads to damage to the growth zones and consecutive growth disturbances [10, 11, 12]. TMJ arthritis can thus result in craniofacial changes, such as micrognathia, retrognathia, and mandibular asymmetry [9, 13, 14]. Contrast‐enhanced MRI is considered the gold standard for detecting early TMJ arthritis, monitoring the disease activity and treatment responses [14]. Additionally, MRI provides long‐term outcome measures such as progression or improvement of bony deformation and mandibular ramus growth [15], based on sequential MR studies conducted at least one year apart to reduce measurement variability.

Longitudinal reference data would facilitate estimating growth potential and treatment response in JIA patients [5]. However, only limited MRI‐based normative reference data are currently available for osseous TMJ components [16, 17].

This study aimed to provide normative osseous measures of TMJs and mandibular ramus according to age and gender and to develop reference data based on a cohort of asymptomatic infants, children, and adolescents.

## 2 Material and Methods

### 2.1 Patients

This cross‐sectional MRI study was approved by the local ethics committee. All participants or their legal guardians provided an informed consent form for using their clinical and imaging data in retrospective analysis [18].

MRI studies of the head, including the TMJs and mandible, performed during a 4‐year period (October 2014 to November 2018), were selected from the picture archiving and communication system (PACS) of a paediatric university hospital.

Our analysis included MRI studies of 133 participants (67 males, 66 females; age range 0–19 years) who were asymptomatic and without JIA diagnosis. From an initial sample of 168 individuals, 35 patients were excluded from further analysis. The exclusion criteria were as follows: (1) the presence of a systemic disease or tumors involving the TMJ, condyle, and masticatory muscles; (2) brain tumors (e.g., craniopharyngioma); (3) pathology of the skull base; (4) insufficient quality images; (5) lack of signed informed consent.

### 2.2 MRI Evaluation

All head MRI studies were performed on a 1.5 Tesla or 3.0 Tesla scanner (Signa Discovery 450 and Discovery 750, GE Medical Systems, Wisconsin, USA) using a head coil according to institutional protocols.

From three dimensional (3D) gradient echo sequences (sagittal acquisition, in plane resolution: 0.5 × 0.5 mm^2^, slice thickness: 0.7–1 mm), axial and sagittal‐oblique images, aligned perpendicular to the long axis of the mandibular condyle and parallel to the mandibular ramus [18], were reconstructed with the multiplanar reconstruction (MPR) tool on the PACS workstation (IDS7, Sectra Medical Systems, Linköping, Sweden).

All measurements were performed on the left and right sides of each patient by a postgraduate trainee (MM). The mean of both sides was calculated for each individual and used for further analysis. To assess inter‐ and intra‐rater reliability, a second postgraduate trainee (JZS) repeated the quantitative measurements on 80 TMJs from 40 randomly selected patients. The two postgraduate trainees, each with three years of experience, were instructed in performing the linear and angle measurements by an experienced radiologist with 20 years of expertise (CJK).

**FIGURE 1:**
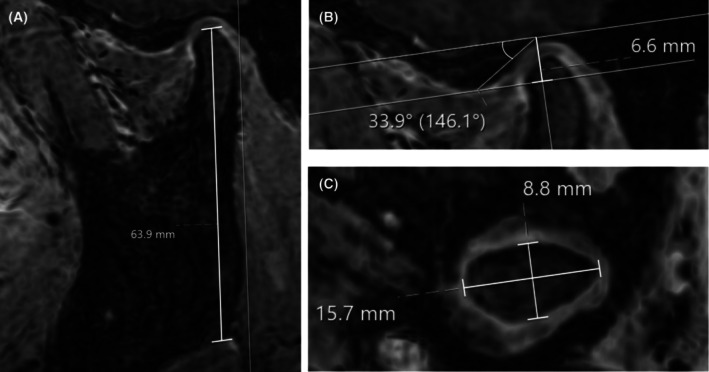
Measurements of osseous temporomandibular joint structures on magnetic resonance images. (A) Ramus height measured on sagittal‐oblique minimum intensity projection (minIP) image. (B) Glenoid fossa depth and articular eminence inclination angle measured on sagittal‐oblique multiplanar reconstruction (MPR) image. (C) Condylar width and depth of the mandibular condyle measured on axial MPR image from a 3D gradient echo sequence.

The ramus height was measured on sagittal‐oblique minimum intensity projection (MinIP) images from the 3D gradient echo sequence on a line parallel to the posterior border of the ramus, from the most cranial point of the condyle to the intersection with the inferior border of the mandibular ramus [6, 19] (Figure 1A). Subvolume MinIP images with 8 mm thickness were constructed in a sagittal‐oblique plane along the long axis of the mandibular ramus and in the coronoid process plane, including the most cranial point of the condyle (condylion) and the most caudal point of the ramus (gonion).

The glenoid fossa depth was measured on sagittal‐oblique MPR images, from the apex of the articular eminence to a horizontal line passing through the upper border of the external auditory canal and the deepest point of the fossa (Modified Frankfurter Line) [18] (Figure 1B).

The articular eminence inclination angle was constructed on sagittal‐oblique MPR images between the Modified Frankfurter Line and a line from the deepest point of the fossa to the apex of the articular eminence [18] (Figure 1B).

The dimensions of the mandibular condyle were measured on axial MPR images (Figure 1C). The condylar width was defined as the distance between the medial‐most and the lateral‐most cortex of the condyle in the axial images in the MRI cross‐section with the biggest area of both condyles [16]. The condylar depth was evaluated as the distance between the anterior‐most and the posterior‐most cortex of the condyle in the axial MRI cross‐section with the largest area of both condyles [16].

### 2.3 Statistical Analysis

To assess the reliability of temporomandibular joint morphology measurements and model the age‐related percentile curves, three key statistical analyses were performed: intra‐rater reliability, inter‐rater reliability, and growth curve modeling.

Intra‐rater reliability was evaluated using measurements obtained by a single measurer (JZS), who recorded two independent observations per image on 80 TMJs of 40 randomly selected patients. A two‐way mixed‐effects single‐measurement absolute agreement intraclass correlation coefficient (ICC) was calculated for each target vector. Additionally, Bland–Altman plots were used to assess and visualize agreement between repeated measurements.

Inter‐rater reliability was assessed by comparing the measurements of two independent measurers, JZS and MM, with the averaged observations from JZS of the 40 patients. The two‐way random‐effects single‐measurement absolute agreement ICC was used to quantify agreement, and Bland–Altman plots were generated to evaluate potential systematic differences between raters.

Age‐related growth curves were estimated for five target vectors (ramus height, glenoid fossa depth, articular eminence inclination angle, condylar width, and condylar depth) using the Box‐Cox Cole and Green (BCCG) distribution, a flexible parametric approach commonly applied in paediatric growth modelling [20]. The three distributional parameters—median, coefficient of variation, and skewness—were modelled as cubic spline functions of age, with the constraint that the median remained non‐decreasing. Optimal degrees of freedom for the splines were selected using the Bayesian information criterion. Centile curves were then derived for each target vector. Since the BCCG‐based quantile equations lacked closed‐form solutions, biannual lookup tables were provided for practical reference (cf. Supplementary Tables 1, 2).

**TABLE 1:** Intra‐observer and inter‐observer reliability of osseous measurements of temporomandibular joint structures on magnetic resonance images.

	Bland–Altman analysis						ICC					
	Intraobserver	Limits of agreement		Interobserver	Limits of agreement		Intraobserver	95% CI		Interobserver	95% CI	
		Lower	Upper		Lower	Upper		Lower	Upper		Lower	Upper
RH	0.07	−1.04	1.17	0.40	−2.95	3.75	1.00	1.00	1.00	0.98	0.96	0.99
CW	0.10	−0.36	0.56	0.48	−1.47	2.44	1.00	0.99	1.00	0.94	0.86	0.97
CD	0.04	−0.36	0.44	0.21	−0.73	1.14	0.98	0.96	0.99	0.87	0.75	0.93
GFD	−0.08	−0.42	0.27	−0.32	−2.03	1.38	0.99	0.98	1.00	0.86	0.73	0.92
AEI	0.17	−1.06	1.40	−0.06	−5.34	5.21	0.99	0.99	1.00	0.89	0.80	0.94

**TABLE 2:** Overview of all measurements of the osseous temporomandibular joint structures and their statistical distribution parameters on magnetic resonance images.

Parameter	Mean		Standard deviation		Median		Minimum		Maximum	
	Male	Female	Male	Female	Male	Female	Male	Female	Male	Female
Ramus height [mm]	48.90	47.74	10.72	10.52	49.55	48.00	24.70	22.80	69.35	44.65
Condylar width [mm]	15.07	14.34	3.04	2.97	14.60	14.3	9.10	9.30	21.40	19.85
Condylar depth [mm]	7.05	6.94	1.12	0.97	6.95	7.00	4.55	5.25	10.20	9.65
Glenoid fossa depth [mm]	6.60	6.25	2.04	1.75	6.50	6.60	1.75	1.95	11.25	9.55
Articular eminence inclination angle [°]	37.71	35.48	6.44	6.55	36.85	35.57	21.15	19.90	50.60	53.25

*Note:* Bland–Altman analysis shows the bias (mm) and the lower and upper limits of agreement (mm) for intra‐ and inter‐observer reliability for each measurement of the TMJ structure. ICC values represent the intra‐ and inter‐observer reliability for each measurement of the TMJ structure.

Abbreviations: AEI, articular eminence inclination angle; CD, condylar depth; CW, condylar width; GFD, glenoid fossa depth; RH, ramus height.

For comparison, a Gaussian approach was also applied, modelling 𝜇(𝑡) and 𝜎(𝑡) under a normal distribution assumption to produce Z‐curves for each target vector.

All analyses were carried out using R 4.1.0. [21], along with the *tidyverse* [22], *blandr* [23], *psych* [24], and *gamlss* [25] packages. The results were considered statistically significant for *p*‐values < 0.05.

## 3 Results

All measurements showed excellent intra‐observer reliability (ICC > 0.98). Ramus height and condylar width measurements showed excellent inter‐observer reliability, while glenoid fossa depth, articular eminence inclination angle, and condylar depth measurements showed good inter‐observer reliability (ICC data and Bland–Altman data for intra‐observer and inter‐observer reliability are given in Table 1).

**FIGURE 2:**
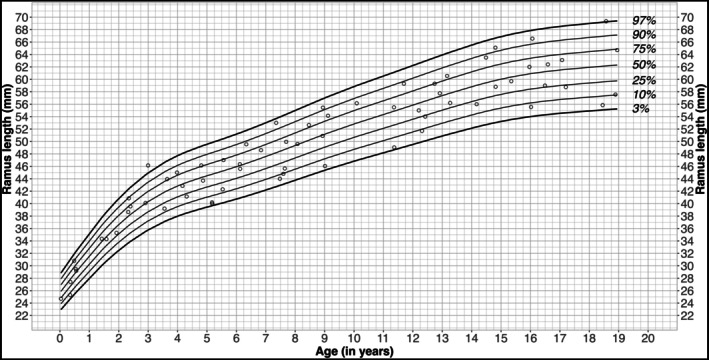
Age related ramus height percentiles for males. Percentiles of the ramus height for males showing the 3rd, 10th, 25th, 50th, 75th, 90th and 97th percentile from 0 to 19 years of age.

**FIGURE 3:**
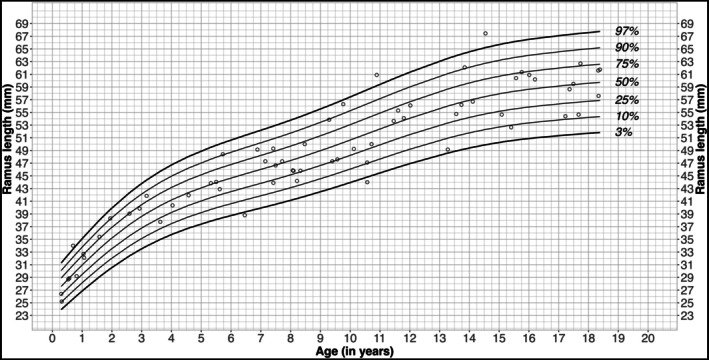
Age related ramus height percentiles for females. Percentiles of the ramus height for females showing the 3rd, 10th, 25th, 50th, 75th, 90th and 97th percentile from 0 to 19 years of age.

The mean ramus height in males was 48.9 mm (SD: 10.72 mm; median: 49.55 mm (IQR: 41.73–56.88 mm)), with a minimum of 24.7 mm and a maximum of 69.35 mm (range: 44.65 mm) (Figure 2, Table 2). In females, the mean ramus height was 47.74 mm (SD: 10.52 mm; median: 48.00 mm (IQR: 41.88–55.9 mm)), with a minimum of 22.80 mm and a maximum of 67.45 mm (range: 44.65 mm) (Figure 3, Table 2). The ramus height showed an age‐related variation with an initial steep increase during early childhood (ages 0–4 years), a second slight increase between the ages of 7 and 14 in females and 7 and 15 in males, followed by a slower but constant increase until the final years of puberty.

**FIGURE 4:**
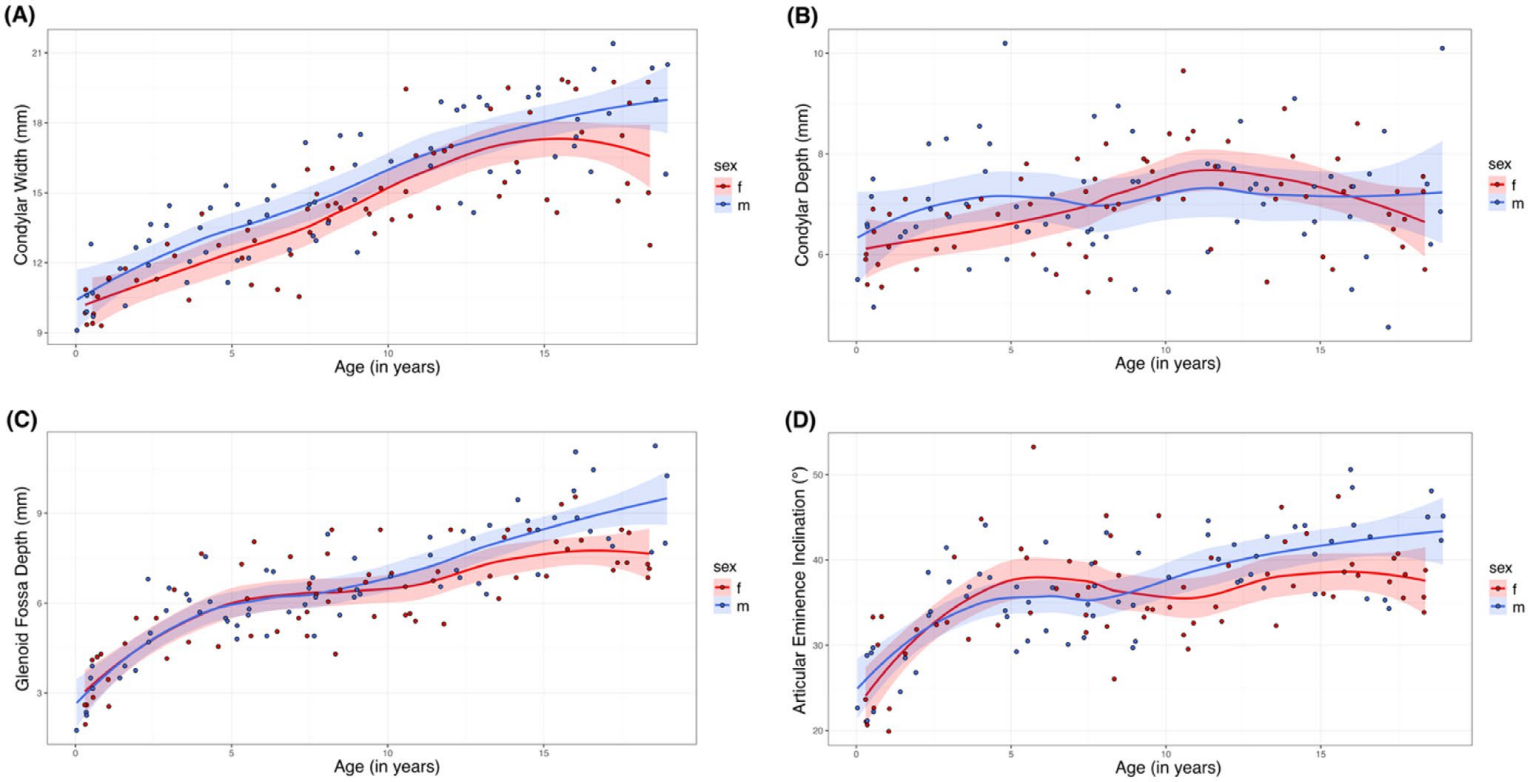
Age‐related spline fits illustrating the changes in osseous temporomandibular joint structures for male (blue) and female (red), with shaded areas representing 95% confidence intervals. (A) Spline fits for condylar width (mm). (B) Spline fits for condylar depth (mm). (C) Spline fits for glenoid fossa depth (mm). (D) Spline fits for articular eminence inclination (°).

The mean condylar width in males was 15.07 mm (SD: 3.04 mm; median: 14.6 mm (IQR: 12.73–17.43 mm)) with a minimum of 9.10 mm and a maximum of 21.40 mm (range: 12.30 mm). The mean condylar width in females measured 14.34 mm (SD: 2.97 mm; median: 14.3 mm (IQR: 12.23–16.24 mm)) with a minimum of 9.30 mm and a maximum of 19.85 mm (range 10.55 mm) (Figure 4A, Table 2). The mean condylar depth for males was 7.05 mm (SD: 1.12 mm; median: 6.95 mm (IQR: 6.45–7.58 mm)) with a minimum of 4.55 mm and a maximum of 10.20 mm (range: 5.65 mm) and for females 6.94 mm (SD: 0.97 mm; median: 7.00 mm (IQR: 6.11–7.54 mm)) with a minimum of 5.25 mm, and a maximum of 9.65 mm (range: 4.4 mm) (Figure 4B, Table 2). The condylar depth showed an age‐related variation, beginning with an initial increase during early childhood, followed by variability throughout adolescence, with males showing a tendency to plateau and females experiencing a slight decrease in late adolescence.

The mean glenoid fossa depth was 6.59 mm in males (SD: 2.04 mm; median: 6.5 mm (IQR: 5.55–8.08 mm)) with a minimum of 1.75 mm and a maximum of 11.25 mm (range 9.50 mm). In females, the mean glenoid fossa depth measured 6.25 mm (SD: 1.76 mm; median: 6.6 mm (IQR: 5.11–7.50 mm)) with a minimum of 1.95 mm and a maximum of 9.55 mm (range: 7.60 mm) (Figure 4C, Table 2). The average articular eminence inclination angle in males measured 36.71° (SD: 6.44°; median: 36.85° (IQR: 33.40°–41.95°)) with a minimum of 21.12° and a maximum of 50.60° (range: 29.45°). In females, the mean articular eminence inclination angle was 35.48° (SD: 6.55°; median: 35.67° (IQR: 32.36°–39.65°)) with a minimum of 19.90° and a maximum of 53.25° (range: 33.35°) (Figure 4D, Table 2).

## 4 Discussion

The present study provided normative, age‐related linear and angular measures of the TMJ and mandibular ramus during the first 19 years of life obtained from three‐dimensional MRI data. Reference standards for age‐related mandibular dimensions in developing individuals are presented.

Mandibles in both females and males demonstrated age‐related variations in posterior height from 0 to 19 years, with a relatively steep increase in the first five years of life, followed by a slower steady increase until the end of puberty. Markic et al. compared different imaging procedures for examining the mandibular ramus height of eight cadaver heads, concluding that there was no significant difference in measuring mandibular ramus height by MRI and CT and suggesting using radiation‐free MRI to follow condylar growth quantitatively by measuring ramus height [6]. Longitudinal studies of children with JIA have shown higher sensitivity of MRI over clinical evaluation in detecting growth of the mandibular ramus following systemic treatment [19] or corticosteroid injection [13]. Bollhalder et al. suggested that systemic treatment of TMJ arthritis in children with JIA maintains normal growth of the mandibular ramus, reduces inflammatory changes seen on MR images, and preserves osseous TMJ morphology [19].

Age‐related dimensions of the mandibular condyle are assumed to include a continuous increase in its size across all directions. However, only a few studies have been conducted regarding the physiological size of the mandibular condyle in children. Christiansen et al. were among the first to describe the size and shape of the mandibular condyle in adult patients using CT scans [26, 27]. Meng et al. demonstrated differences in TMJ morphology between child cadavers and adult volunteers [28]. Karlo et al. evaluated the size and shape of the mandibular condyle in asymptomatic subjects using CT scans in the transverse imaging plane [29]. Comparing our results with the diagrams provided by Karlo et al., a similar course of age‐associated condylar depth and width can be demonstrated, suggesting that there is no need to use ionising radiation to measure osseous TMJ structures.

Two different methods are known to measure the articular eminence inclination angle. In the best‐fit line method, the angle is measured between the best‐fit line on the posterior slope of the articular eminence and the Frankfurter Line. The top‐roof line method measures the angle between the Frankfurter Line and a line passing through the deepest point of the fossa to the apex of the articular eminence [30]. Katsavrias et al. suggested that the top‐roof line focuses on the location of the apex of the articular eminence relative to the roof of the fossa, which is affected by the development of the eminence height. In contrast, the best‐fit line focuses on the posterior surface of the eminence, representing the actual condylar path. Our study used the top‐roof line method because it better represents the articular eminence morphology and is more suited for evaluating the TMJ morphology. The present findings generally agree with the study from Nickel et al. [31], where the TMJ eminence has more than 50% of its mature size by age three with a corresponding similar increase of glenoid fossa depth and articular eminence inclination angle.

Deformations of the mandibular condyles are common in elderly patients as a result of age‐related osseous degeneration [32]; however, erosions and osseous deformations are increasingly prevalent in the TMJ of paediatric patients with JIA [10]. Contrast‐enhanced MRI remains the only imaging method for detecting early TMJ arthritis, monitoring the inflammatory disease activity and treatment response for reaching treatment decisions [14]. However, for measuring the dimensions of osseous structures contrast‐enhancement is not required. Optimal and accurate visualisation and evaluation of osseous structures by MRI are achieved with 3D gradient echo or 3D ultrashort echo time sequences, available on any MR system including the evolving dental‐dedicated MRI (ddMRI) systems. Most existing evidence for diagnosing TMJ disorders using MRI is based on scanners operating at 1.5 Tesla and 3.0 Tesla. However, Nixdorf et al. reported no significant differences in TMJ diagnostic accuracy at 0.55 and 1.5 Tesla [33]. Further research is needed to determine whether the ramus height can be reliably assessed by ddMRI systems.

Numerous CT studies have demonstrated the skeletal morphology and development of TMJs [26, 27, 28, 29]. Previous MRI studies examining large samples of TMJs with normal appearance have primarily focused on evaluating inflammatory joint components, including joint and bone marrow enhancement [34, 35, 36]. To our knowledge, no extensive studies have provided normative data on skeletal parameters measured by MRI of the TMJs and mandibular ramus in asymptomatic children and adolescents. The age‐related ramus height percentiles for males and females, derived from the cross‐sectional measurements in this study, are the first to visualize the age‐related height of the mandibular ramus from infancy to adolescence, offering a valuable framework for assessing ramus height changes in children. The percentile diagrams can be applied in clinical practice to assess ramus height in successive MRI studies.

### 4.1 Limitations

Two postgraduate trainees independently performed the MRI measurements following initial training and calibration from an experienced radiologist. The calibration process included reviewing measurement definitions, landmark identification, and plane alignment protocols to minimise measurement variability. The potential for examiner‐related bias could affect the robustness of the results, but the intra‐ and inter‐observer reliability was good to excellent for all measurements.

Although the use of multiple MRI scanners could have introduced variability in image quality, the consistent spatial resolution of the MRI sequence across scanners suggests that the linear distance measurements were unlikely to be affected. Nonetheless, other potential sources of variability should still be considered when interpreting the results.

A cross‐sectional study design provides a practical way to generate normative data across different age groups. However, this approach does not capture intra‐individual growth patterns and may be influenced by differences in pubertal timing, potentially leading to misestimations of growth spurts and peak velocity. These variabilities should be carefully considered when making clinical decisions, particularly in planning growth modification treatments for patients with skeletal Class II malocclusions. In contrast, a longitudinal study design could capture the pubertal growth spurt more accurately through serial measurements, despite the range of pubertal onset observed in cross‐sectional data. Nonetheless, repeated MRI scans in healthy children raise ethical concerns, particularly regarding the need for sedation in younger children. Additionally, obtaining normative data would require studying individuals with asymptomatic TMJ who are undergoing head MRIs for other medical reasons, as a longitudinal study of asymptomatic children from infancy to adolescence is ethically unfeasible.

Although natural asymmetry in mandibular ramus height is well documented in both healthy individuals and patients with JIA, the right and left side measurements were averaged to improve measurement accuracy instead of assessing asymmetry. Each side was measured three times by the postgraduate trainees (twice by JZS and once by MM), but these repeated measures were not independent and therefore could not be included separately in the estimation process. Averaging these measurements refined the true value by minimising random error. While side‐specific differences are clinically relevant, this approach prioritised generating reliable overall values, and future studies may address asymmetry using statistical models that consider intra‐subject dependence.

## 5 Conclusions

This study provides age‐related normative osseous measures of the TMJs and mandibular ramus in asymptomatic infants, children, and adolescents aged 0–19 years. Additionally, age‐related percentile curves for mandibular ramus height are presented, which can be used to assess the growth of the mandibular ramus in children with JIA in order to monitor and manage disease activity and treatment response in clinical practice.

## Author Contributions

**Juliet Zuying Shen:** Investigation, data curation, writing – original draft, writing – review and editing. **Maurice Molnar:** Conceptualisation, methodology, validation, investigation, data curation. **Dominik Alois Ettlin:** Conceptualisation, methodology, validation, writing – review and editing. **Daniel Beat Wiedemeier:** Methodology, validation, formal analysis, data curation, writing – review and editing, visualisation. **Christian Johannes Kellenberger:** Conceptualisation, methodology, validation, formal analysis, resources, writing – review and editing, supervision. All authors reviewed and approved the final version of the manuscript.

## Ethics Statement

This study was approved by the Swiss Ethics Committees (BASEC), approval number 2018–00094.

## Conflicts of Interest

The authors declare no conflicts of interest.

## Supporting information


**Supplementary Table 1** Biannual lookup table of ramus height values (in mm) in males showing percentiles (ranging from 2.5th to the 97.5th) from 0 to 19 years of age.

**Supplementary Table 2** Biannual lookup table of ramus height values (in mm) in females showing percentiles (ranging from 2.5th to the 97.5th) from 0 to 19 years of age.

## Data Availability

The data that support the findings of this study are available from the corresponding author upon reasonable request.
