# Supplementary material for: Age‐Related Osseous Measures of the Temporomandibular Joint and Mandibular Ramus During Childhood Assessed by Magnetic Resonance Imaging
Source: Orthod Craniofac Res. 2025 Dec 17;29(2):300–8. doi: 10.1111/ocr.70071 (PMC12972259; doi:10.1111/ocr.70071)
Supplement: Supplementary file 1 — Supplementary Table 1 Biannual lookup table of ramus height values (in mm) in males showing percentiles (ranging from 2.5th to the 97.5th) from 0 to 19 years of age. [file OCR-29-300-s002.pdf]

| age | percentile 2.5th | 3rd | 5th | 10th | 15th | 20th | 25th | 30th | 35th | 40th | 45th | 50th | 55th | 60th | 65th | 70th | 75th | 80th | 85th | 90th | 95th | 97th | 97.5th |
|---|---|---|---|---|---|---|---|---|---|---|---|---|---|---|---|---|---|---|---|---|---|---|---|
| 1 | 27.849 | 27.999 | 28.447 | 29.137 | 29.603 | 29.973 | 30.291 | 30.577 | 30.842 | 31.094 | 31.338 | 31.577 | 31.817 | 32.061 | 32.313 | 32.579 | 32.866 | 33.186 | 33.559 | 34.028 | 34.725 | 35.177 | 35.329 |
| 1.5 | 30.234 | 30.397 | 30.883 | 31.632 | 32.138 | 32.54 | 32.886 | 33.196 | 33.484 | 33.757 | 34.021 | 34.282 | 34.542 | 34.807 | 35.081 | 35.369 | 35.681 | 36.028 | 36.433 | 36.942 | 37.698 | 38.19 | 38.355 |
| 2 | 32.303 | 32.477 | 32.996 | 33.797 | 34.337 | 34.767 | 35.137 | 35.468 | 35.776 | 36.067 | 36.35 | 36.628 | 36.906 | 37.189 | 37.482 | 37.79 | 38.123 | 38.494 | 38.926 | 39.471 | 40.279 | 40.804 | 40.98 |
| 2.5 | 34.07 | 34.254 | 34.801 | 35.645 | 36.216 | 36.669 | 37.058 | 37.408 | 37.732 | 38.04 | 38.338 | 38.631 | 38.925 | 39.223 | 39.532 | 39.857 | 40.208 | 40.599 | 41.055 | 41.63 | 42.482 | 43.036 | 43.221 |
| 3 | 35.571 | 35.762 | 36.334 | 37.216 | 37.811 | 38.284 | 38.691 | 39.056 | 39.394 | 39.716 | 40.027 | 40.333 | 40.64 | 40.951 | 41.273 | 41.613 | 41.979 | 42.388 | 42.864 | 43.464 | 44.353 | 44.931 | 45.125 |
| 3.5 | 36.79 | 36.989 | 37.58 | 38.492 | 39.107 | 39.597 | 40.017 | 40.395 | 40.745 | 41.078 | 41.399 | 41.716 | 42.033 | 42.355 | 42.688 | 43.039 | 43.418 | 43.841 | 44.333 | 44.954 | 45.874 | 46.472 | 46.673 |
| 4 | 37.777 | 37.981 | 38.588 | 39.524 | 40.156 | 40.659 | 41.091 | 41.479 | 41.838 | 42.179 | 42.51 | 42.835 | 43.16 | 43.491 | 43.833 | 44.194 | 44.583 | 45.017 | 45.523 | 46.16 | 47.104 | 47.718 | 47.925 |
| 4.5 | 38.559 | 38.766 | 39.386 | 40.342 | 40.987 | 41.5 | 41.941 | 42.337 | 42.704 | 43.052 | 43.389 | 43.721 | 44.053 | 44.391 | 44.74 | 45.108 | 45.505 | 45.948 | 46.464 | 47.114 | 48.079 | 48.705 | 48.916 |
| 5 | 39.231 | 39.443 | 40.073 | 41.046 | 41.702 | 42.224 | 42.672 | 43.075 | 43.449 | 43.803 | 44.146 | 44.484 | 44.822 | 45.165 | 45.521 | 45.895 | 46.299 | 46.75 | 47.275 | 47.936 | 48.918 | 49.555 | 49.769 |
| 5.5 | 39.859 | 40.074 | 40.715 | 41.702 | 42.369 | 42.9 | 43.355 | 43.765 | 44.144 | 44.504 | 44.853 | 45.196 | 45.539 | 45.888 | 46.249 | 46.63 | 47.04 | 47.498 | 48.032 | 48.704 | 49.7 | 50.348 | 50.566 |
| 6 | 40.517 | 40.736 | 41.387 | 42.391 | 43.069 | 43.608 | 44.071 | 44.487 | 44.873 | 45.239 | 45.593 | 45.942 | 46.291 | 46.646 | 47.013 | 47.399 | 47.817 | 48.282 | 48.825 | 49.508 | 50.521 | 51.18 | 51.401 |
| 6.5 | 41.22 | 41.442 | 42.104 | 43.126 | 43.816 | 44.364 | 44.835 | 45.258 | 45.651 | 46.023 | 46.384 | 46.738 | 47.094 | 47.454 | 47.828 | 48.221 | 48.646 | 49.119 | 49.671 | 50.366 | 51.397 | 52.067 | 52.292 |
| 7 | 41.963 | 42.189 | 42.863 | 43.903 | 44.605 | 45.164 | 45.643 | 46.074 | 46.473 | 46.853 | 47.22 | 47.581 | 47.942 | 48.31 | 48.69 | 49.09 | 49.523 | 50.004 | 50.566 | 51.274 | 52.323 | 53.005 | 53.234 |
| 7.5 | 42.737 | 42.967 | 43.654 | 44.713 | 45.428 | 45.997 | 46.485 | 46.924 | 47.331 | 47.717 | 48.091 | 48.459 | 48.827 | 49.201 | 49.588 | 49.996 | 50.436 | 50.927 | 51.499 | 52.22 | 53.289 | 53.983 | 54.216 |
| 8 | 43.533 | 43.767 | 44.467 | 45.546 | 46.274 | 46.854 | 47.351 | 47.798 | 48.212 | 48.606 | 48.986 | 49.361 | 49.736 | 50.117 | 50.511 | 50.927 | 51.375 | 51.875 | 52.458 | 53.192 | 54.281 | 54.988 | 55.226 |
| 8.5 | 44.332 | 44.571 | 45.284 | 46.382 | 47.124 | 47.714 | 48.221 | 48.676 | 49.098 | 49.499 | 49.886 | 50.268 | 50.65 | 51.038 | 51.439 | 51.863 | 52.319 | 52.828 | 53.422 | 54.169 | 55.278 | 55.999 | 56.241 |
| 9 | 45.119 | 45.362 | 46.088 | 47.206 | 47.961 | 48.561 | 49.077 | 49.54 | 49.969 | 50.377 | 50.772 | 51.16 | 51.549 | 51.944 | 52.352 | 52.783 | 53.248 | 53.766 | 54.37 | 55.131 | 56.259 | 56.993 | 57.239 |
| 9.5 | 45.865 | 46.112 | 46.85 | 47.986 | 48.754 | 49.364 | 49.888 | 50.359 | 50.796 | 51.21 | 51.611 | 52.006 | 52.401 | 52.803 | 53.218 | 53.656 | 54.128 | 54.655 | 55.269 | 56.042 | 57.189 | 57.935 | 58.185 |
| 10 | 46.582 | 46.833 | 47.582 | 48.736 | 49.515 | 50.135 | 50.668 | 51.146 | 51.589 | 52.01 | 52.417 | 52.818 | 53.22 | 53.628 | 54.049 | 54.494 | 54.974 | 55.509 | 56.132 | 56.918 | 58.083 | 58.84 | 59.094 |
| 10.5 | 47.264 | 47.519 | 48.279 | 49.45 | 50.241 | 50.87 | 51.41 | 51.895 | 52.345 | 52.772 | 53.185 | 53.592 | 53.999 | 54.413 | 54.841 | 55.292 | 55.779 | 56.322 | 56.955 | 57.752 | 58.934 | 59.702 | 59.96 |
| 11 | 47.923 | 48.181 | 48.952 | 50.139 | 50.941 | 51.579 | 52.127 | 52.619 | 53.075 | 53.508 | 53.927 | 54.339 | 54.752 | 55.172 | 55.606 | 56.063 | 56.557 | 57.107 | 57.749 | 58.557 | 59.755 | 60.534 | 60.796 |
| 11.5 | 48.585 | 48.847 | 49.628 | 50.832 | 51.645 | 52.291 | 52.846 | 53.345 | 53.808 | 54.247 | 54.671 | 55.09 | 55.508 | 55.934 | 56.374 | 56.837 | 57.338 | 57.896 | 58.546 | 59.365 | 60.58 | 61.37 | 61.635 |
| 12 | 49.259 | 49.524 | 50.316 | 51.537 | 52.361 | 53.017 | 53.579 | 54.085 | 54.554 | 54.999 | 55.43 | 55.854 | 56.278 | 56.709 | 57.155 | 57.626 | 58.133 | 58.699 | 59.358 | 60.189 | 61.421 | 62.221 | 62.49 |
| 12.5 | 49.936 | 50.205 | 51.007 | 52.245 | 53.08 | 53.745 | 54.316 | 54.828 | 55.304 | 55.755 | 56.191 | 56.621 | 57.051 | 57.489 | 57.941 | 58.417 | 58.932 | 59.505 | 60.174 | 61.016 | 62.265 | 63.076 | 63.349 |
| 13 | 50.604 | 50.877 | 51.69 | 52.944 | 53.791 | 54.465 | 55.043 | 55.562 | 56.044 | 56.501 | 56.944 | 57.379 | 57.815 | 58.258 | 58.716 | 59.199 | 59.721 | 60.302 | 60.98 | 61.833 | 63.098 | 63.921 | 64.197 |
| 13.5 | 51.248 | 51.525 | 52.348 | 53.618 | 54.476 | 55.158 | 55.743 | 56.27 | 56.757 | 57.22 | 57.669 | 58.11 | 58.551 | 59 | 59.464 | 59.953 | 60.481 | 61.07 | 61.756 | 62.62 | 63.901 | 64.734 | 65.014 |
| 14 | 51.866 | 52.145 | 52.979 | 54.264 | 55.132 | 55.822 | 56.415 | 56.948 | 57.441 | 57.91 | 58.363 | 58.81 | 59.257 | 59.711 | 60.18 | 60.675 | 61.21 | 61.805 | 62.5 | 63.374 | 64.671 | 65.514 | 65.798 |
| 14.5 | 52.437 | 52.72 | 53.563 | 54.862 | 55.74 | 56.437 | 57.037 | 57.575 | 58.074 | 58.548 | 59.006 | 59.458 | 59.91 | 60.369 | 60.843 | 61.344 | 61.884 | 62.486 | 63.188 | 64.072 | 65.384 | 66.236 | 66.522 |
| 15 | 52.939 | 53.225 | 54.076 | 55.387 | 56.273 | 56.978 | 57.583 | 58.126 | 58.63 | 59.109 | 59.572 | 60.027 | 60.483 | 60.947 | 61.426 | 61.931 | 62.477 | 63.085 | 63.794 | 64.686 | 66.01 | 66.871 | 67.16 |
| 15.5 | 53.368 | 53.656 | 54.514 | 55.836 | 56.729 | 57.44 | 58.049 | 58.597 | 59.105 | 59.587 | 60.054 | 60.514 | 60.973 | 61.441 | 61.924 | 62.433 | 62.983 | 63.596 | 64.31 | 65.21 | 66.545 | 67.412 | 67.704 |
| 16 | 53.718 | 54.008 | 54.871 | 56.202 | 57.101 | 57.816 | 58.43 | 58.981 | 59.493 | 59.978 | 60.448 | 60.91 | 61.373 | 61.843 | 62.33 | 62.842 | 63.396 | 64.013 | 64.732 | 65.638 | 66.981 | 67.854 | 68.147 |
| 16.5 | 54.003 | 54.294 | 55.162 | 56.5 | 57.403 | 58.122 | 58.739 | 59.294 | 59.808 | 60.296 | 60.768 | 61.233 | 61.698 | 62.171 | 62.66 | 63.175 | 63.732 | 64.352 | 65.075 | 65.985 | 67.336 | 68.214 | 68.508 |
| 17 | 54.238 | 54.531 | 55.403 | 56.747 | 57.654 | 58.376 | 58.996 | 59.553 | 60.069 | 60.559 | 61.033 | 61.5 | 61.968 | 62.442 | 62.933 | 63.451 | 64.01 | 64.633 | 65.359 | 66.273 | 67.63 | 68.512 | 68.808 |
| 17.5 | 54.44 | 54.733 | 55.608 | 56.957 | 57.868 | 58.593 | 59.215 | 59.774 | 60.292 | 60.784 | 61.26 | 61.729 | 62.198 | 62.674 | 63.167 | 63.687 | 64.248 | 64.873 | 65.602 | 66.519 | 67.881 | 68.766 | 69.063 |
| 18 | 54.623 | 54.917 | 55.795 | 57.149 | 58.063 | 58.79 | 59.414 | 59.975 | 60.495 | 60.988 | 61.466 | 61.936 | 62.407 | 62.885 | 63.379 | 63.901 | 64.464 | 65.091 | 65.822 | 66.743 | 68.109 | 68.997 | 69.295 |
| 18.5 | 54.797 | 55.093 | 55.973 | 57.331 | 58.248 | 58.978 | 59.604 | 60.166 | 60.688 | 61.183 | 61.662 | 62.134 | 62.606 | 63.086 | 63.582 | 64.105 | 64.67 | 65.299 | 66.032 | 66.956 | 68.327 | 69.217 | 69.516 |
| 19 | 54.957 | 55.253 | 56.136 | 57.498 | 58.418 | 59.149 | 59.777 | 60.341 | 60.864 | 61.361 | 61.841 | 62.315 | 62.788 | 63.269 | 63.767 | 64.291 | 64.858 | 65.489 | 66.224 | 67.151 | 68.525 | 69.419 | 69.719 |

**Supplementary table 1** Biannual lookup table of ramus height values (in mm) in males showing percentiles (ranging from 2.5th to the 97.5th) from 0 to 19 years of age.
