# Supplementary material for: Age‐Related Osseous Measures of the Temporomandibular Joint and Mandibular Ramus During Childhood Assessed by Magnetic Resonance Imaging
Source: Orthod Craniofac Res. 2025 Dec 17;29(2):300–8. doi: 10.1111/ocr.70071 (PMC12972259; doi:10.1111/ocr.70071)
Supplement: Supplementary file 2 — Supplementary Table 2 Biannual lookup table of ramus height values (in mm) in females showing percentiles (ranging from 2.5th to the 97.5th) from 0 to 19 years of age. [file OCR-29-300-s001.pdf]

age	percentile 2.5th	3rd	5th	10th	15th	20th	25th	30th	35th	40th	45th	50th	55th	60th	65th	70th	75th	80th	85th	90th	95th	97th	97.5th
1	26.678	26.849	27.359	28.147	28.679	29.103	29.467	29.795	30.099	30.387	30.667	30.942	31.217	31.498	31.787	32.093	32.423	32.792	33.221	33.763	34.567	35.09	35.266
1.5	28.604	28.787	29.334	30.178	30.749	31.204	31.594	31.945	32.271	32.581	32.88	33.175	33.471	33.771	34.082	34.41	34.764	35.159	35.619	36.2	37.062	37.623	37.812
2	30.363	30.557	31.138	32.034	32.64	33.122	33.537	33.91	34.255	34.584	34.902	35.215	35.529	35.847	36.177	36.525	36.901	37.32	37.809	38.426	39.341	39.937	40.137
2.5	31.923	32.128	32.738	33.68	34.317	34.824	35.26	35.652	36.016	36.361	36.695	37.024	37.354	37.689	38.036	38.402	38.797	39.238	39.752	40.4	41.362	41.989	42.199
3	33.3	33.513	34.15	35.132	35.797	36.326	36.781	37.19	37.569	37.929	38.278	38.621	38.965	39.315	39.677	40.058	40.471	40.93	41.466	42.142	43.146	43.799	44.019
3.5	34.491	34.712	35.371	36.389	37.077	37.625	38.096	38.52	38.912	39.285	39.647	40.002	40.359	40.721	41.096	41.491	41.918	42.394	42.95	43.65	44.689	45.366	45.593
4	35.524	35.752	36.431	37.48	38.189	38.753	39.238	39.674	40.079	40.463	40.835	41.201	41.568	41.941	42.327	42.734	43.174	43.664	44.237	44.958	46.029	46.725	46.96
4.5	36.414	36.647	37.343	38.418	39.145	39.723	40.22	40.667	41.082	41.476	41.857	42.233	42.609	42.991	43.387	43.804	44.255	44.758	45.344	46.083	47.181	47.895	48.135
5	37.182	37.421	38.131	39.229	39.971	40.562	41.069	41.526	41.949	42.351	42.741	43.124	43.508	43.899	44.303	44.729	45.189	45.702	46.301	47.056	48.177	48.906	49.151
5.5	37.876	38.119	38.843	39.961	40.717	41.318	41.836	42.3	42.732	43.141	43.538	43.929	44.32	44.718	45.129	45.563	46.032	46.555	47.165	47.934	49.076	49.819	50.068
6	38.501	38.748	39.484	40.62	41.389	42	42.526	42.999	43.437	43.853	44.257	44.654	45.051	45.456	45.874	46.315	46.792	47.323	47.944	48.725	49.886	50.641	50.895
6.5	39.098	39.349	40.096	41.25	42.031	42.652	43.186	43.666	44.111	44.534	44.943	45.347	45.75	46.161	46.586	47.034	47.518	48.058	48.687	49.481	50.66	51.427	51.684
7	39.679	39.933	40.692	41.863	42.655	43.285	43.827	44.314	44.766	45.195	45.61	46.02	46.429	46.846	47.277	47.732	48.223	48.771	49.41	50.215	51.412	52.19	52.451
7.5	40.269	40.527	41.297	42.485	43.289	43.929	44.479	44.973	45.432	45.867	46.289	46.704	47.12	47.543	47.981	48.442	48.941	49.497	50.145	50.963	52.176	52.966	53.232
8	40.888	41.15	41.932	43.138	43.955	44.604	45.162	45.664	46.13	46.572	47	47.422	47.844	48.274	48.718	49.187	49.693	50.257	50.916	51.746	52.978	53.78	54.05
8.5	41.538	41.804	42.598	43.824	44.653	45.313	45.88	46.39	46.863	47.312	47.747	48.176	48.605	49.041	49.492	49.968	50.483	51.056	51.725	52.568	53.82	54.635	54.909
9	42.222	42.493	43.3	44.546	45.389	46.06	46.636	47.155	47.635	48.092	48.534	48.97	49.406	49.849	50.308	50.792	51.315	51.897	52.577	53.434	54.707	55.535	55.814
9.5	42.93	43.205	44.026	45.293	46.15	46.832	47.418	47.945	48.434	48.898	49.348	49.791	50.234	50.685	51.151	51.643	52.175	52.767	53.459	54.33	55.624	56.466	56.749
10	43.668	43.948	44.783	46.071	46.943	47.637	48.233	48.769	49.266	49.738	50.196	50.646	51.097	51.556	52.03	52.531	53.071	53.674	54.377	55.264	56.58	57.437	57.724
10.5	44.419	44.704	45.553	46.864	47.75	48.456	49.063	49.608	50.114	50.594	51.059	51.518	51.976	52.443	52.925	53.434	53.984	54.597	55.313	56.215	57.554	58.425	58.718
11	45.17	45.46	46.324	47.657	48.558	49.276	49.893	50.447	50.961	51.45	51.923	52.389	52.855	53.33	53.821	54.338	54.898	55.521	56.249	57.166	58.527	59.413	59.711
11.5	45.911	46.205	47.083	48.438	49.355	50.084	50.711	51.274	51.797	52.294	52.774	53.248	53.722	54.204	54.703	55.229	55.798	56.432	57.171	58.103	59.487	60.388	60.69
12	46.629	46.927	47.819	49.195	50.126	50.867	51.503	52.076	52.606	53.111	53.599	54.08	54.562	55.051	55.558	56.092	56.67	57.313	58.064	59.011	60.416	61.331	61.638
12.5	47.298	47.601	48.506	49.902	50.846	51.597	52.243	52.824	53.362	53.874	54.369	54.857	55.345	55.842	56.356	56.898	57.484	58.137	58.898	59.858	61.284	62.212	62.524
13	47.943	48.251	49.167	50.582	51.539	52.301	52.956	53.544	54.09	54.608	55.111	55.605	56.1	56.604	57.125	57.674	58.268	58.93	59.702	60.675	62.12	63.061	63.376
13.5	48.544	48.855	49.783	51.215	52.184	52.956	53.618	54.214	54.767	55.292	55.8	56.301	56.803	57.312	57.84	58.396	58.997	59.667	60.449	61.434	62.898	63.85	64.17
14	49.076	49.39	50.329	51.777	52.757	53.536	54.206	54.809	55.368	55.898	56.412	56.919	57.425	57.941	58.474	59.036	59.644	60.321	61.112	62.108	63.587	64.55	64.873
14.5	49.546	49.863	50.811	52.273	53.262	54.049	54.725	55.333	55.898	56.433	56.952	57.464	57.975	58.496	59.034	59.602	60.215	60.899	61.697	62.703	64.196	65.168	65.495
15	49.946	50.266	51.221	52.695	53.692	54.486	55.168	55.781	56.349	56.89	57.413	57.928	58.444	58.968	59.511	60.083	60.702	61.391	62.196	63.209	64.715	65.695	66.024
15.5	50.283	50.605	51.566	53.05	54.054	54.853	55.539	56.156	56.729	57.273	57.799	58.318	58.837	59.366	59.912	60.488	61.111	61.805	62.615	63.635	65.151	66.137	66.469
16	50.562	50.886	51.853	53.345	54.355	55.158	55.848	56.469	57.045	57.591	58.121	58.643	59.165	59.696	60.245	60.825	61.451	62.149	62.963	63.989	65.513	66.505	66.838
16.5	50.796	51.122	52.093	53.592	54.606	55.413	56.107	56.73	57.309	57.858	58.39	58.914	59.439	59.972	60.524	61.106	61.735	62.436	63.254	64.285	65.817	66.813	67.148
17	51.007	51.333	52.309	53.814	54.832	55.642	56.339	56.965	57.546	58.097	58.631	59.158	59.684	60.22	60.774	61.359	61.99	62.694	63.516	64.551	66.089	67.089	67.426
17.5	51.2	51.528	52.507	54.018	55.04	55.854	56.552	57.181	57.764	58.318	58.854	59.382	59.911	60.449	61.005	61.592	62.226	62.932	63.757	64.796	66.34	67.344	67.681
18	51.377	51.706	52.689	54.205	55.23	56.047	56.748	57.379	57.964	58.519	59.057	59.587	60.118	60.658	61.216	61.805	62.441	63.15	63.977	65.02	66.569	67.577	67.915
18.5	51.549	51.879	52.865	54.386	55.415	56.234	56.938	57.571	58.158	58.715	59.255	59.787	60.319	60.861	61.421	62.011	62.65	63.361	64.191	65.238	66.792	67.803	68.143
19	51.72	52.051	53.04	54.566	55.599	56.42	57.127	57.761	58.35	58.91	59.451	59.985	60.519	61.062	61.624	62.217	62.857	63.571	64.404	65.454	67.013	68.027	68.368

Supplementary table 2 Biannual lookup table of ramus height values (in mm) in females showing percentiles (ranging from 2.5th to the 97.5th) from 0 to 19 years of age.
